# Liver Transplantation Despite Active Intracranial Hemorrhage in Acetaminophen‐Induced Acute Liver Failure: A Case Report

**DOI:** 10.1002/ccr3.73432

**Published:** 2026-09-02

**Authors:** Daryna Shnitser, Eslam Fouda, Ramona Nicolau‐Raducu, Fouad G. Souki

**Affiliations:** ^1^ University of South Carolina School of Medicine Greenville USA; ^2^ Department of Anesthesiology University of Miami, Jackson Memorial Hospital Miami USA

**Keywords:** acetaminophen toxicity, acute liver failure, intracranial hemorrhage, liver transplantation, neurocritical care

## Abstract

Acute liver failure with intracranial hemorrhage poses difficult transplant decisions. We report acetaminophen‐induced acute liver failure with cerebral edema and active multifocal hemorrhage successfully managed with emergent liver transplantation and staged neurosurgical intervention. This case supports individualized transplantation when hepatic prognosis is fatal and neurologic recovery remains plausible.

## Key Clinical Message

Active intracranial hemorrhage should not automatically preclude liver transplantation in selected acute liver failure patients when hepatic prognosis is otherwise fatal, neurologic injury appears potentially reversible, and perioperative neuroprotection with neurosurgical rescue is available.

## 1 Introduction

Acute liver failure (ALF) is an uncommon but life‐threatening syndrome defined by abrupt severe hepatic dysfunction in individuals without preexisting liver disease, typically characterized by coagulopathy and hepatic encephalopathy within 26 weeks of illness onset [1, 2]. In The United States, ALF affects approximately 2000 patients annually, with drug‐induced liver injury accounting for a substantial proportion of cases and acetaminophen toxicity representing the leading etiology [3, 4]. Because ALF may progress rapidly to multiorgan failure, cerebral edema, and death, early transfer to a transplant‐capable center is central to management [2, 5, 6, 7].

Liver transplantation (LT) remains the definitive rescue therapy for selected patients unlikely to recover spontaneously. Prognostic systems such as the King's College Criteria and urgency frameworks such as United Network for Organ Sharing (UNOS) Status 1 designation help identify emergency candidates, but decisions require integration of physiologic deterioration, neurologic status, and anticipated reversibility rather than reliance on any single score [2, 4, 8, 9]. Intracranial hemorrhage (ICH) is an uncommon but devastating complication in ALF, reported in approximately 1%–5% of cases. Although bleeding complications in ALF are less common than laboratory coagulopathy alone might suggest, intracranial bleeding remains uniquely consequential because it threatens neurologic recovery and perioperative safety [10, 11]. Its occurrence creates a major management dilemma: proceeding with transplantation may expose the patient to hemorrhage progression during major surgery, whereas deferring transplantation in severe ALF may eliminate the only realistic chance of survival [10].

Evidence guiding transplant eligibility and perioperative neurosurgical management in ALF with active ICH remains limited, and decisions depend on multidisciplinary assessment of hepatic prognosis, neurologic reversibility, and institutional rescue capacity [10, 12, 13]. We describe a case of acetaminophen‐induced ALF complicated by hepatic encephalopathy, cerebral edema, and multifocal ICH, successfully managed with emergent LT followed by staged neurosurgical intervention. Written informed consent was obtained from the patient.

## 2 Case History

### 2.1 Preoperative Course

A 45‐year‐old woman with acute liver failure and circulatory shock was transferred to our medical center for urgent liver transplant evaluation. One week earlier, she had presented to an outside hospital with fever, headache, epigastric pain, and malaise, and was discharged after leukopenia and mild transaminitis were attributed to a presumed viral illness. She returned 2 days later with nausea, vomiting, syncope, and fluid‐responsive hypotension. She denied alcohol or illicit drug use, recent travel, and sick contacts. Medication history revealed acetaminophen ingestion of 4–6 g/day over several days for postoperative pain following abdominoplasty and breast augmentation 6 weeks earlier.

Emergency department evaluation demonstrated severe hepatocellular injury, thrombocytopenia, and coagulopathy, with mild to moderate ascites on abdominal computed tomography (CT). Laboratory values included AST > 7500 U/L, ALT 3741 U/L, lactate 5.2 mmol/L, ammonia < 9 μmol/L, INR 2.3, creatinine 0.91 mg/dL, hemoglobin 12.3 g/dL, and platelets 8000/mm^3^.

She was admitted to the intensive care unit (ICU) with fulminant hepatic failure, intubated, and started on N‐acetylcysteine, broad‐spectrum antimicrobials, corticosteroids, plasmapheresis, intravenous immunoglobulin, and lactulose. Her course was complicated by vasopressor‐dependent shock, transfusion‐requiring coagulopathy, renal failure requiring continuous venovenous hemodialysis (CVVHD), and recurrent hypoglycemia. She was transferred to our transplant center on ICU day 2, where infectious causes of liver injury were excluded.

On ICU day 4, she was listed as Status 1A for liver transplantation with an estimated 21‐day transplant‐free survival of 6%, based on AST > 3000 U/L, age > 18 years, ALF, ICU admission, mechanical ventilation, renal replacement therapy, and INR > 2.0. Initial brain CT was unremarkable; repeat imaging on ICU day 4 revealed multiple bilateral frontoparietal intraparenchymal hemorrhages at the gray‐white matter junction with mild surrounding edema and no midline shift (largest measuring 3.3 cm in the left parietal lobe). Neurosurgical management recommendations included hypertonic saline, levetiracetam, active correction of coagulopathy, head elevation, controlled hyperventilation, target serum sodium levels of 145–155 mmol/L, target systolic blood pressure of 140–160 mmHg, and avoidance of hyperthermia and fluid overload.

**FIGURE 1:**
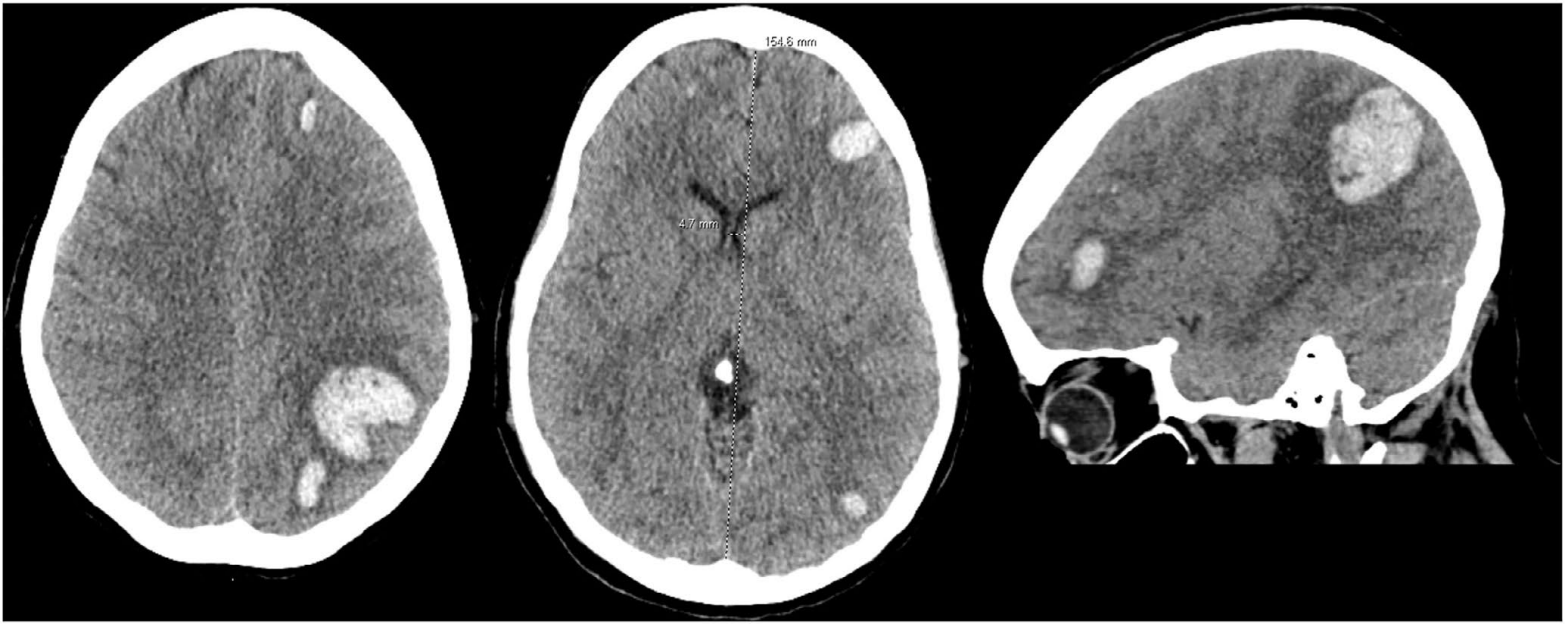
Pretransplant brain CT demonstrating multifocal intraparenchymal hemorrhages with rightward midline shift.

On ICU day 5, a suitable liver from a donor after brain death became available. Repeat head CT demonstrated interval enlargement of multifocal intraparenchymal hemorrhages with new mass effect and a 4.7 mm rightward midline shift (Figure 1). Despite these high‐risk findings, her clinical neuro‐examination demonstrated preserved pupillary (equal and reactive to light) and brainstem reflexes without signs of uncal or transtentorial herniation. She maintained stable physiological responses to neuroprotective measures, achieving target serum hypernatremia (145–155 mmol/L). Following urgent multidisciplinary consultation among transplant surgery, hepatology, neurocritical care, and neurosurgery, consensus was reached to proceed with emergent transplantation. The team concluded that reversing her end‐stage acute hepatic failure (MELD‐Na 35, Status 1A)—the primary etiology of her severe coagulopathy—offered the only viable chance to halt ICH progression, whereas withholding transplantation portended 100% short‐term mortality without guaranteeing neurological stabilization.

### 2.2 Intraoperative Course

**FIGURE 2:**
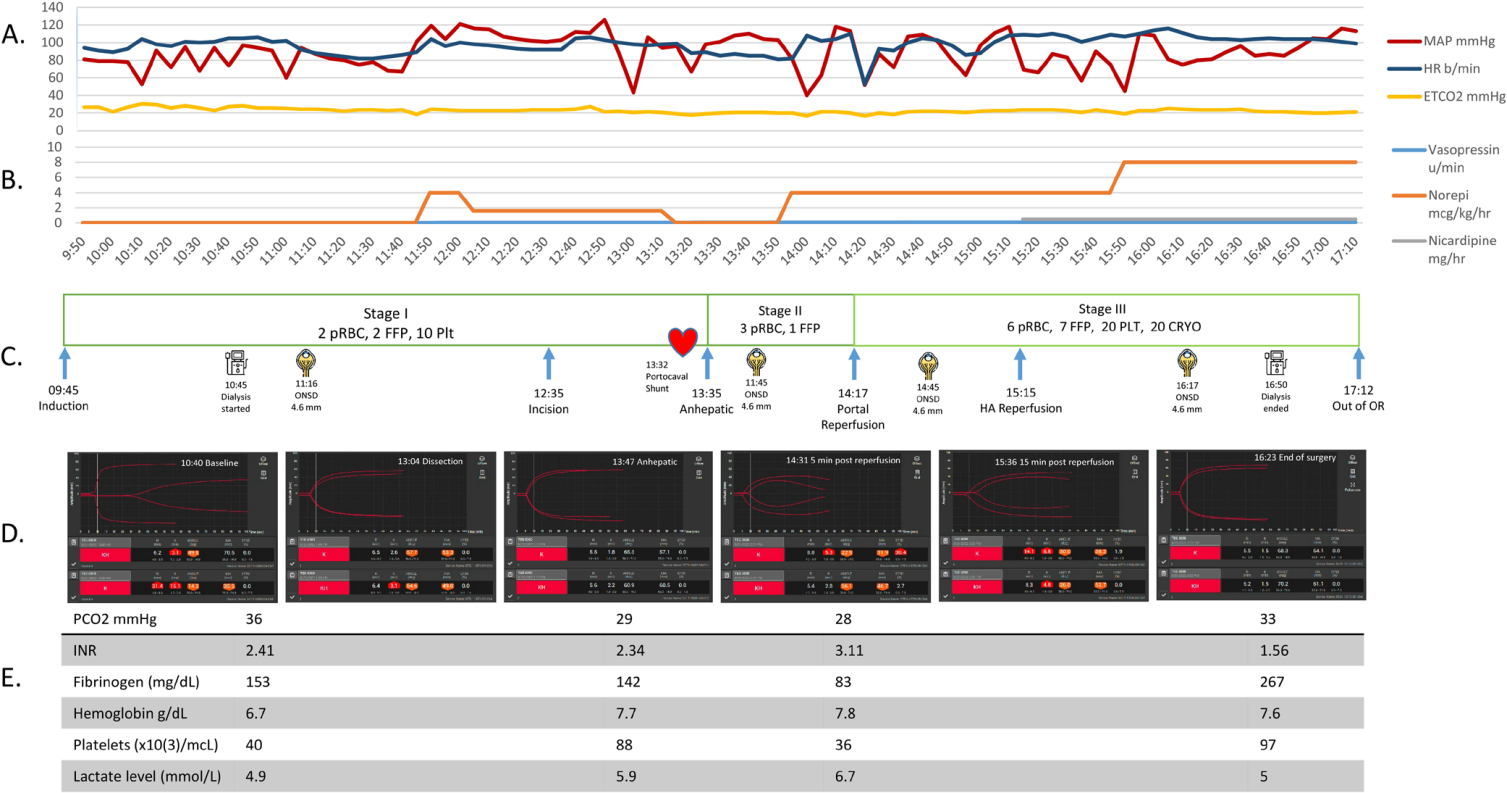
Intraoperative thromboelastography and hemodynamics management. (A) Intraoperative hemodynamics and tracing of mean arterial pressure [MAP], heart rate [HR], and end tidal CO_2_ [ETCO_2_]; (B) Intraoperative management of vasopressors and nicardipine; (C) Measurement of ONSD: Optic nerve sheath diameter by ultrasound and intraoperative coagulopathy correction during different stages of surgery [stage 1: Dissection phase, stage 2: Anhepatic phase, Stage 3: Neohepatic phase] with pRBC: Packed red blood cells, FFP: Fresh frozen plasma; PLT: Platelets; and CRYO: Cryoprecipitate; (D) Serial intraoperative Thromboelastography [TEG] as baseline, dissection, anhepatic, 5 min post portal reperfusion, post hepatic artery (HA) reperfusion, and at end of surgery; (E) Intraoperative laboratory tests at baseline, during anhepatic and neohepatic phases.

Anesthesia was maintained with sevoflurane, fentanyl, cisatracurium, and propofol. Vascular access consisted of 9F and 12F multilumen internal jugular catheters, a 12F femoral venous catheter, and bilateral radial arterial lines. CVVHD was run at 250 mL/min via femoral outflow and jugular inflow cannulas, also serving as partial venovenous bypass during transplant surgery. Hemodynamics were monitored with transesophageal echocardiography and FloTrac/Vigileo. Coagulation was assessed with thromboelastography (TEG) and standard laboratory testing, with markedly abnormal baseline TEG (Figure 2).

A targeted neuroprotective strategy was implemented to limit hemorrhage expansion and cerebral edema, including head elevation, controlled hyperventilation with PaCO_2_ < 30 mmHg, mannitol 1 g/kg, strict hemodynamic control using norepinephrine, vasopressin, and nicardipine, correction of coagulopathy, and avoidance of volume overload through ultrafiltration. Systolic blood pressure was maintained above 100 mmHg throughout. Levetiracetam was continued, and serial ultrasound optic nerve sheath diameter measurements remained stable at 4–5 mm.

A piggyback technique with temporary portocaval shunt was used during hepatectomy to enhance hemodynamic stability and reduce blood loss. Total operative time was 4.5 h, with a warm ischemia time of 23 min and cold ischemia time of 5 h 22 min. Intraoperatively, she received 2.5 L crystalloid, 1 L 5% albumin, 11 units packed red blood cells, 10 units fresh frozen plasma, 30 units platelets, and 20 units cryoprecipitate. Estimated blood loss was 5 L, with 2 L removed via CVVHD. She was transferred intubated to the ICU for close neurologic monitoring.

### 2.3 Outcome and Follow‐Up

Explant pathology confirmed acetaminophen‐induced hepatic necrosis, with approximately 70% hepatocyte loss involving zones 2 and 3. Postoperative CT of the brain demonstrated progression of intracerebral hemorrhage with increased edema and midline shift to 8 mm. Fifteen hours after transplantation, neurosurgical intervention was undertaken with placement of a fiberoptic intracranial pressure monitor, which showed an opening pressure of 27 cm H_2_O and an external ventricular drain.

Following these interventions, the hemorrhages stabilized, with gradual improvement in cerebral edema and neurologic examination. She was extubated on postoperative day (POD) 7. The external ventricular drain was removed on POD 9, and renal replacement therapy was discontinued on POD 14. Her later course included partial right internal jugular vein thrombosis on POD 18, transfer to rehabilitation on POD 30, brief readmission for new‐onset seizure on POD 32, and discharge home on POD 65. At the one‐ and two‐year post‐transplant follow‐ups, her neurological examination was completely normal. She was alert and fully oriented, with preserved speech, language, attention, and executive function. Motor, sensory, coordination, and gait assessments were unremarkable. Overall, these findings demonstrated a complete return to baseline neurological function and functional independence, allowing her to resume part‐time employment.

## 3 Discussion

### 3.1 Transplant Timing and Selection in ALF With Active Intracranial Hemorrhage

The primary challenge was not recognizing the need for LT but determining whether and when to proceed amid multiorgan failure and evolving structural brain injury. Within days, she progressed to fulminant hepatic failure with shock, renal replacement therapy, mechanical ventilation, and profound thrombocytopenic coagulopathy, making spontaneous hepatic recovery exceedingly unlikely [4, 5]. The predicted 21‐day transplant‐free survival of 6% and MELD‐Na of 35 framed LT as the only realistic life‐saving option.

Her transfer and early listing were consistent with AASLD, ACG, and EASL guidance that ALF patients be managed in an intensive care setting at a transplant‐capable center, with continuous reassessment for transplantation as clinical status evolves [2, 6, 7, 14, 15]. Although prognostic systems such as King's College Criteria remain central, contemporary guidance emphasizes that no single score should be used in isolation and that listing decisions should incorporate serial deterioration, multiorgan failure, and dynamic candidacy assessment [2, 6, 9].

The emergence and enlargement of multifocal frontoparietal intraparenchymal hemorrhages with developing midline shift introduced a parallel neurocritical dilemma. Because standard hepatic prognostic tools do not incorporate ICH, candidacy relied on individualized multidisciplinary judgment rather than a validated algorithm [2, 10]. The decision to proceed reflected two converging conclusions: survival without transplantation was highly unlikely, and the pattern of brain injury still allowed a plausible chance of meaningful recovery [12, 16].

Active ICH has traditionally been viewed as a major adverse prognostic factor and, in some settings, as a relative or absolute contraindication to LT because intraoperative hemodynamic shifts, severe coagulopathy, and massive transfusion requirements may exacerbate bleeding [2, 10, 11]. Rather than eliminating this concern, our case refines it. Alongside emerging reports of successful transplantation despite severe intracranial bleeding, it suggests that candidacy should depend on whether the hemorrhage pattern, neurological examination, radiographic trajectory, and response to neuroprotective measures preserve a plausible path toward meaningful recovery once hepatic failure is reversed [13].

In our patient, although repeat imaging demonstrated progressive mass effect with a 4.7 mm midline shift, the hemorrhage pattern was predominantly localized to the gray–white matter junction without brainstem destruction. Furthermore, her intact brainstem reflexes and stable ONSD measurements (4–5 mm) demonstrated preserved neurovascular compensation under active neuroprotective protocols. Recognizing that uncorrected acute liver failure and profound coagulopathy would inevitably lead to fatal intracranial expansion, the multidisciplinary team determined that emergent transplantation was not only justifiable, but represented the definitive intervention required to arrest neurological deterioration.

### 3.2 Neurosurgical Thresholds and Their Modification in ALF

Neurosurgical decision‐making for ICH in patients without liver disease is guided by radiographic and clinical thresholds. For acute subdural hematomas, evacuation is generally recommended when thickness exceeds 10 mm or midline shift exceeds 5 mm, or when acute deterioration is attributable to the collection [17]. For intraparenchymal hemorrhage, surgical evacuation is typically considered with significant mass effect or neurologic decline, cerebellar hemorrhage larger than 3 cm, refractory intracranial hypertension, or superficial lobar hemorrhage causing treatable mass effect [18, 19]. These criteria assume preserved coagulation and platelet function. In ALF, severe thrombocytopenia, labile coagulopathy, hyperfibrinolysis, and impaired wound healing shift the risk–benefit calculus for invasive neurosurgical intervention [10, 20].

In this patient, multifocal supratentorial hemorrhages with evolving mass effect might have triggered earlier surgical consideration with normal hemostasis, but profound thrombocytopenia and coagulopathy favored initial medical management while transplantation was organized. Hyperosmolar therapy, strict blood pressure and sodium control, head elevation, controlled ventilation, seizure prophylaxis, and serial imaging were used to limit secondary injury. TEG‐guided correction with platelets, plasma, and cryoprecipitate supported transplantation and optimized conditions for later neurosurgical procedures [10, 21]. Only after partial normalization of hemostasis was it reasonable to proceed with intracranial pressure monitoring and external ventricular drainage.

### 3.3 Peri‐Transplant Management of Intracranial Hemorrhage

The peri‐transplant strategy addressed a unique precarious physiology. In ALF, coagulopathy, thrombocytopenia, hyperfibrinolysis, hyperammonemia, endothelial dysfunction, and systemic inflammation may increase susceptibility to intracranial bleeding and hematoma propagation [2, 14, 20]. Loss of cerebral autoregulation may also render the cerebral circulation pressure‐passive, making both hypotension and blood pressure surges potentially harmful [19, 22]. Management therefore required correction of coagulopathy, preservation of cerebral perfusion, limitation of intracranial hypertension, and avoidance of abrupt osmotic or hemodynamic shifts.

From an anesthetic and critical care standpoint, a deliberately neuroprotective strategy was used. Tight arterial pressure control with vasoactive infusions was guided by advanced hemodynamic monitoring, consistent with contemporary neurocritical care and transplant recommendations [14, 22]. Continuous renal replacement therapy permitted close control of volume status and electrolytes, limiting both fluid overload and abrupt osmotic shifts; current acute liver failure guidance supports early continuous hemofiltration to help modulate ammonia and limit worsening encephalopathy and cerebral edema [15]. Ventilation was adjusted to maintain PaCO_2_ in the low‐normal range, and osmotherapy with mannitol, along with high‐normal serum sodium targets, was used to mitigate intracranial hypertension [2, 14]. Serial ultrasound optic nerve sheath diameter measurements provided a noninvasive surrogate for intracranial pressure trends while invasive monitoring was deferred because of coagulopathy, aligning with published data supporting this approach in high‐risk patients and in patients with intracranial hemorrhage or severe brain injury [23, 24].

The piggyback technique with temporary portocaval shunt likely contributed to more stable macrohemodynamics during the anhepatic phase. By preserving venous return and attenuating abrupt increases in central venous pressure, the shunt may have helped maintain cerebral perfusion pressure and reduce venous congestion‐driven rises in intracranial pressure. Temporary portocaval shunting has been associated with improved hemodynamic stability, preserved renal function, and reduced transfusion requirements in liver transplantation [25]. TEG‐guided transfusion further helped balance hemorrhagic and thrombotic risks [11, 20, 21].

### 3.4 Postoperative Neurosurgical Intervention and Neurologic Recovery

Postoperative neuroimaging confirmed progression of ICH, with increased edema and midline shift to 8 mm. By that time, partial correction of coagulopathy permitted safer placement of a parenchymal intracranial pressure monitor and external ventricular drain. This staged approach reduced procedural bleeding risk while enabling definitive management of intracranial hypertension [10]. Following ventriculostomy and intracranial pressure‐directed therapy, the hemorrhages stabilized, cerebral edema improved, and the neurologic examination recovered sufficiently to permit extubation on POD 7. Later discontinuation of renal replacement therapy, discharge home, and normal neurologic examination at 2 years support the principle that neurologic dysfunction in ALF may be reversible when the metabolic insult is corrected before herniation or irreversible structural injury occurs [12, 14, 16].

This case illustrates the clinical and ethical tension introduced by active ICH in an otherwise transplant‐eligible patient with ALF. Proceeding with transplantation risks intraoperative hemorrhagic progression, severe neurologic injury, and possible futile use of a donor organ; deferring transplantation in otherwise irreversible ALF may effectively guarantee death from progressive multisystem organ failure [10, 12]. The favorable outcome here, together with recently reported successful transplantation after recurrent ICH, suggests that active ICH should not be treated as an automatic exclusion from transplantation in every patient with ALF [13]. Instead, decisions should integrate hepatic prognosis without transplantation, neurologic reversibility, feasibility of perioperative neuroprotection, and institutional capacity for timely neurosurgical intervention.

Several limitations temper interpretation. As a single case report, this experience cannot establish the safety, efficacy, or generalizability of LT in ALF complicated by active ICH. The favorable outcome may reflect patient‐specific factors, including hemorrhage location, absence of brainstem injury or herniation, timely transfer, donor availability, and institutional expertise. The contribution of each intervention cannot be isolated because hemostatic correction, neuroprotective anesthesia, renal replacement therapy, osmotherapy, transplantation, and staged neurosurgical intervention occurred as an integrated rescue strategy. Thus, the case does not define a new algorithm but highlights a framework for selected patients in whom hepatic prognosis is otherwise fatal and neurologic injury remains potentially reversible.

## 4 Conclusion

Acute liver failure complicated by active intracranial hemorrhage presents a high‐stakes conflict between hepatic urgency and neurologic risk. This case demonstrates that intracranial hemorrhage should not necessarily be viewed as an automatic barrier to liver transplantation when neurologic injury appears potentially reversible and intensive perioperative neuroprotection and neurosurgical rescue are available. Successful management requires rapid transfer, dynamic reassessment of transplant candidacy, individualized evaluation of neurologic reversibility, and tightly coordinated hepatology, transplant surgery, anesthesia, critical care, and neurosurgical decision‐making. Selected patients with fatal acute liver failure may achieve meaningful recovery when transplantation and staged neurologic intervention are integrated within a multidisciplinary rescue strategy.

## Author Contributions

**Eslam Fouda:** writing – original draft, data curation. **Fouad G. Souki:** conceptualization, writing – original draft, writing – review and editing, data curation, supervision. **Daryna Shnitser:** writing – original draft, writing – review and editing. **Ramona Nicolau‐Raducu:** conceptualization, data curation, supervision, writing – original draft, writing – review and editing.

## Funding

The authors have nothing to report.

## Ethics Statement

Ethics approval was not required for this single‐patient case report according to institutional policy.

## Consent

Written informed consent was obtained from the patient for publication of this case report and any accompanying images.

## Conflicts of Interest

The authors declare no conflicts of interest.

## Data Availability

The data that support the findings of this study are available from the corresponding author upon reasonable request.
